# Selective Surface Acoustic Wave-Based Organophosphorus Sensor Employing a Host-Guest Self-Assembly Monolayer of β-Cyclodextrin Derivative

**DOI:** 10.3390/s150817916

**Published:** 2015-07-23

**Authors:** Yong Pan, Ning Mu, Shengyu Shao, Liu Yang, Wen Wang, Xiao Xie, Shitang He

**Affiliations:** 1 State Key Laboratory of NBC Protection for Civilian, Yangfang, Changping District, Beijing 102205, China; E-Mails: Sdmuta@163.com (N.M.); Shaoshengyu@163.com (S.S.); Yangliujinjin@sina.com (L.Y.); 2 Institute of Acoustic, Chinese Academy of Science, Zhongguancun Street, Haidian District, Beijing 100080, China; E-Mails: Wangwenwq@mail.ioa.ac.cn (W.W.); Xiexiao08@mails.ucas.ac.cn (X.X.); Heshitang@ mail.ioa.ac.cn (S.H.)

**Keywords:** surface acoustic wave (SAW), β-cyclodextrin, self-assembly, molecular imprinting (MIP), detection

## Abstract

Self-assembly and molecular imprinting technologies are very attractive technologies for the development of artificial recognition systems and provide chemical recognition based on need and not happenstance. In this paper, we employed a β-cyclodextrin derivative surface acoustic wave (SAW) chemical sensor for detecting the chemical warfare agents (CWAs) sarin (O-Isoprophyl methylphosphonofluoridate, GB). Using sarin acid (isoprophyl hydrogen methylphosphonate) as an imprinting template, mono[6-deoxy-6-[(mercaptodecamethylene)thio]]-β-cyclodextrin was prepared by self-assembled method on one of the SAW oscillators. After templates’ removal, a sensitive and selective molecular imprinting (MIP) monolayer for GB was prepared. Electrochemical impedance spectroscopy and atomic force microscope (AFM) were used to characterize this film. Comparing the detection results to GB by MIP film and non-MIP film, the molecularly imprinting effect was also proved. The resulting SAW sensor could detect sarin as low as 0.10 mg/m^3^ at room temperature and the frequency shift was about 300 Hz. The response frequency increased linearly with increasing sarin concentration in the range of 0.7 mg/m^3^~3.0 mg/m^3^. When sarin was detected under different temperatures, the SAW sensor exhibited outstanding sensitivity and reliability.

## 1. Introduction

Chemical warfare agents (CWAs) are powerful weapons and a threat to civil safety. They are extremely hazardous and potentially lethal. As these agents can be dispersed as a gas, liquid, aerosol or powder, developing methods to detect CWAs at very low limits as quickly as possible is very important to ensure civilian safety [1,2,3]. Interest in surface acoustic wave (SAW) gas sensors have recently grown because of their excellent performance, low cost, short response time, small size, light weight, high reproducibility, and low power dissipation [4,5,6]. Self-assembly films rely on a unique combination of many interactions to produce highly ordered, durable films ranging in thickness from one to tens of molecular layer are an important film preparing technology for SAW sensors. In 1992, Larry J. Kepley published the first report on the combination of SAW technology and self-assembly technology (SAM) to detect the organophosphorus compound dimethyl methyl phosphorate (DMMP) [7]. Because chemisorption of alkanethiol on bare gold can generate densely packed and highly ordered monolayer films with sulfur atoms bound tightly to the surface of gold, and specific co-functionalized alkanethiols can be used to tailor the interfacial properties of the surface, this method is considered an excellent technique for studying the effects of changes in molecular structure and composition on interfacial properties [8].

Molecular imprinting (MIP) is an effective strategy for encoding information on a molecular scale in bulk polymeric or supramolecular materials [9]. In 1998, Dickert published a report on the MIP polymer SAW sensor was used for determing o-dimethyl-benzene, the detection limit of this sensor was 4.5 mg/m^3^ and the device displayed excellent detection effects [10]. The preparation of a MIP film is based on a cross-linking process using functional monomers that have a specific interaction with templates (print molecules). Removal of the template yields a porous structure with cavities that allow spatial arrangements of functional groups corresponding to the template molecules. Therefore, MIP features selectivity for rebinding the template that prints the coating. Different approaches have been made to design sensor layers that are capable of incorporating analytes with high selectivity. The bottleneck in the development of chemical sensors is the design of the coating for chemical recognition of the analyte [11,12,13]. Several pronounced methods have been reported to tailor supramolecular cavities for different analytes. For example, Bing-Qing Cao prepared a supramolecular SAW sensor based on a calixarene derivatives membrane that showed higher sensitivity and selectivity to detect organophosphorus compounds [14].

**Figure 1:**
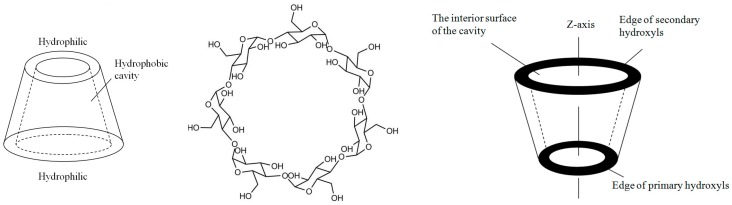
Chemical structure of β-cyclodextrin.

Although functionalized cyclodextrins have been studied for various applications, such as enzyme mimics, separation, and drug delivery [15,16,17], few reports about cyclodextrin as a sensitive film for SAW sensor detection of warfare agents have been published [18]. The host-guest electronic state, steric complementarity, and host preorganization are three key elements for cyclodextrin use in a sensor film. Cyclodextrins have a preorganized rigid hydrophobic cavity of three different sizes and hydroxyl groups at the rims of this cavity that allow for various chemical transformations, Figure 1. The binding characteristics of CDs change upon functionalization with hydroxyl groups because of the resultant changes in the shape, size, and charge distributions of the cavity and the loss of certain intermolecular hydrogen bonds. In this paper, sarin acid was selected as a template modifying β-cyclodextrin through upper and lower rim functionalization, and a SAW sensor with high selectivity and sensitivity toward GB was fabricated.

## 2. Experimental Section

### 2.1. Reagents and Instruments

Mono[6-deoxy-6-[(mercaptodecamethylene)thio]]-β-CD was synthesized according to [19,20,21] in our laboratory and analyzed by TOF-MS, FTIR, and NMR. Isoprophyl methylphosphonofluoridate and isoprophyl hydrogen methylphosphonate were obtained from the Research Institute of Chemical Defense, Beijing, China. 1,10-Decanedithiol (AR, TCI company, Tokyo, Japan), β-CD, and mono (6-O-p-tolylsulfonyl)-β-CD (AR, Shanghai Chemical Reagent, Beijing, China), and all other chemicals used were of reagent grade. A Model Proteck C3100 frequency counter (Proteck Company, Incheon, Korea) was also used.

The vapor source for SAW sensor testing was a FH-10 vapor generator (Research Institute of Chemical Defense, Beijing, China). Vapors were generated as described [22] by bubbling dry N_2_ through a glass container maintained at 20 °C and diluted from near saturation via a pulsed modulation technique.

### 2.2. SAW Resonator and Oscillator Fabrication

Lower insertion loss, higher Q factor, and single resonation mode will significantly improve the frequency stability of the SAW oscillator and improve sensor performance by promoting higher sensitivity and a lower limit of detection. In this section, 150 MHz two-port SAW resonators were fabricated by standard lithographic techniques on the same ST-X quartz substrate [23]. The quartz substrate is well-known for its low temperature coefficient of frequency. The numbers of electrode pairs for the IDT and reflector were 51 and 400, respectively. Between the transducers, a region of 2 mm^2^ gold was inserted for sensitive film deposition. Measurement results obtained by a network analyzer showed good agreement with the simulated one [24].

**Figure 2:**
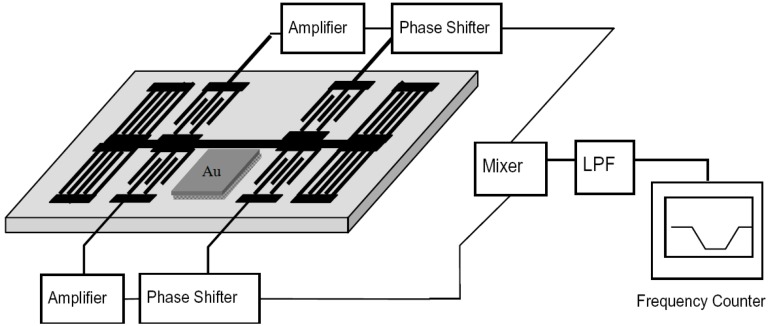
The oscillator circuit configuration.

The output and input transducers of the fabricated 150 MHz SAW resonator were connected by oscillator circuit that was made of discrete elements (*i.e.*, amplifier with a gain of 15 dB, phase shifter, mixer, and LPF) connected to each other by SMA connectors, as shown in Figure 2. The output of the amplifier was mixed to obtain a difference frequency in the MHz range. This technique allows reduction of the influence of thermal expansion of the substrate and use of simple low-frequency counters. The output of the oscillator was connected to a programmable frequency counter.

### 2.3. Preparation of a Self-Assembled, Molecularly Imprinted Film for the SAW Sensor

Before the experiment, the above-mentioned SAW resonators were prepared. First, the Au surface of the SAW film deposition area was purged with a 3:1 (V/V) piranha (sulfuric acid, hydrogen peroxide) solution for the cleansing and brightening, puffed with high purity N_2_ to remove surface foreign substances, and measured to determine its the fundamental frequency. Second, 100 mL of 2 × 10^−4^ M mono[6-deoxy-6-[(mercaptodecamethylene)thio]]-β-CD self-assembly ethanol solution was prepared. A solution of 100 mL 5 × 10^−4^ M isoprophyl hydrogen methylphosphonate template was added to this solution followed by mixing for several minutes. The self-assembly solution was then deposited on the clean Au surface for 24 h for using later. Third, the SAW resonator was immersed into the self-assembly solution for 24 h, retrieved, and washed with ethanol to remove the excess template molecules(isoprophyl hydrogen methylphosphonate). The fundamental frequency and noise were measured again. Results indicated that the self-assembled MIP molecularly imprinted film of mono[6-deoxy-6-[(mercaptodecamethylene)thio]]-β-CD was successfully formed on the Au surface of the SAW resonator.

## 3. Results and Discussion

### 3.1. Calculation for Immobilization of Cyclodextrin Derivative on Gold

Given the high mass-sensitivity of the SAW sensor, the thickness of the self-assembled MIP film was estimated by measuring the frequency shift with Sauerbrey formula as follows [7,12]:

Δf = −1.26 × 10^6^f_0_^2^hρ (1)

where Δf (Hz) is the frequency shift between the coated and uncoated SAW resonators, f_0_ (MHz) is the operating frequency of the SAW sensor, h (cm) is the film thickness of the self-assembled MIP film, and ρ (g/cm^3^) is the density of the film material.

The frequency of the SAW sensor was set to 150 MHz, the density of mono[6-deoxy-6-[(mercaptodecamethylene)thio]]-β-CD was 1.5 g/cm^3^, and Δf was measured to be 10–15 kHz; thus, the thickness of self-assembled film was estimated to be 2–4 nm on average.

### 3.2. Analysis by AFM and Electrochemical Impedance Spectroscopy

**Figure 3:**
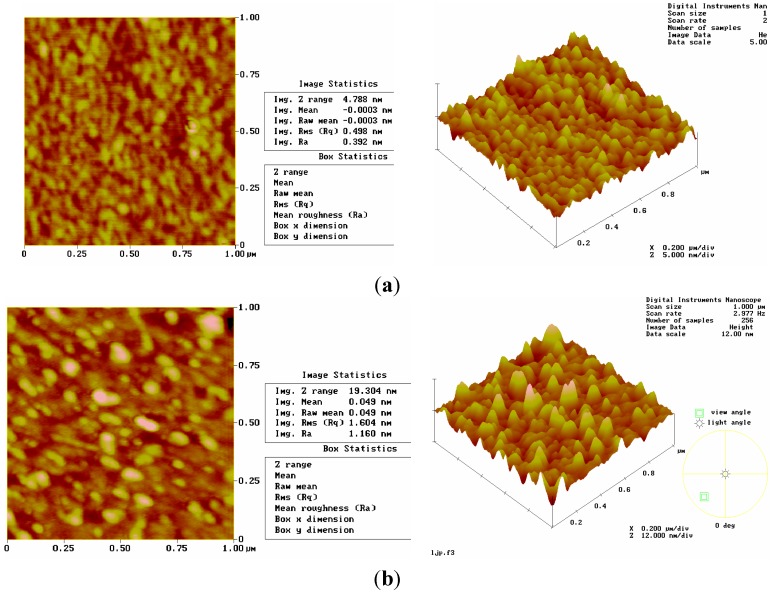
Atomic force microscope (AFM) two-dimensional and three-dimensional photographs of the surface acoustic wave (SAW) Au surface. (**a**) Bare Au film; (**b**) After the self-assembly procedure.

Atomic force microscopy（AFM） and electrochemical impedance spectroscopy (EIS) are often used to examine the characterisctics of the film surface. Change in the film surface can be seen in Figure 3. When the Au coating area of the SAW oscillator is not coated with mono[6-deoxy-6-[(mercaptodecamethylene)thio]]-β-CD, the RMS[Rq] of Au is 0.498 nm, and its surface is orderly; Upon self-assembly of the MIP film, however, RMS[Rq] rises to 1.604 nm, which is about 3 times that of Au, so the Au surface becomes much rougher. This result reveals that a self-assembled film has formed on the Au coating area. At same time, the 3D structures also illustrate the difference between the uncoated and the coated Au.

**Figure 4:**
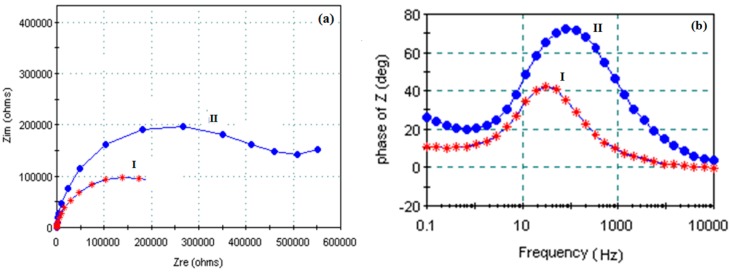
Electrochemical impedance spectra (**a**) Nyquist; (**b**) Bode Phase.

Analysis through EIS also proves the formation of the self-assembled film, Figure 4a,b. The impedance of the Au film is small (I). When self-assembly of the mono[6-deoxy-6-[(mercaptodecamethylene)thio]]-β-CD film is completed on the Au area, because of the existence of coating, the impedance increased significantly (II).

### 3.3. Confirmation of Molecular Imprinting Effect

**Figure 5:**
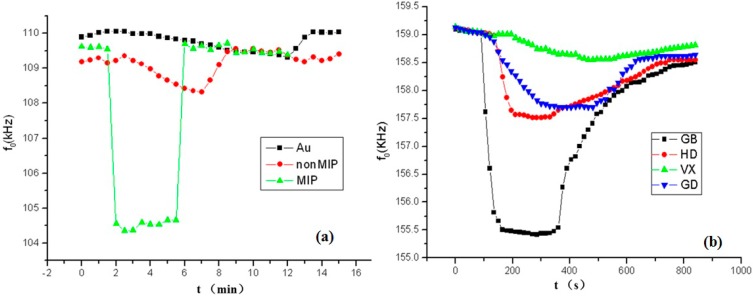
Confirmation of MIP effect (**a**) Detection of GB by three kinds of sensors; (**b**) Detection of different warfare agents by MIP sensor.

To prove the imprinting effect, GB was detected at the same concentration (7.5 mg/m^3^) with three other SAW sensors. First, the SAW gold area was not coated with the MIP film, and GB was detected only with bare Au. No interaction occurs between the Au film and GB molecules, so a frequency shift is not observed. Second, GB was detected with the self-assembled film without templates (SAW-nonMIP sensor). Although some adsorption occurs between the film and the GB molecules, the lack of imprinting effects of the film inhibited viewing of the frequency shift. Finally, when GB is detected with the self-assembled SAW-MIP sensor, a high frequency, short response time, and low recovery speed are obtained because of the imprinting effect (Figure 5a).

To illustrate the imprinting effect further, GB, O-pinacolyl methylphosphonofluoridate (GD), O-ethyl-S-(β-diisopropylaminioethyl)methylphosphonothiolate (VX), and Bis(2-chloroethyl)sulfide (HD) were detected under the same conditions (5.5 mg/m^3^) with the SAW-MIP sensor. As the 3D structure of sarin acid is similar to that of GB, GB molecules can easily enter the sensor cavities and combine with the imprinted film. As such, the response signal is very obvious, and both the response time and recovery time are very short. When GD, VX, and HD are detected with the same SAW-MIP sensor, however, significant difference were observed, 3D structures of VX and HD are very different from that of GB. Thus, VX and HD are difficult to immerse into the imprinting cavities and the adsorption between the film and the CWA molecules is weak. The response signals, response times, and recovery speeds of VX and HD are very different from those of GB. By comparing these results, the imprinting effect is confirmed (Figure 5b).

### 3.4. Detection of Different Concentrations of Sarin with the SAW-MIP Sensor

Being a kind of mass sensor, the acoustic wave velocity and the frequency of the SAW-MIP sensor are shifted when a change of mass happens on the surface of the sensitive layer due to the sorption of volatiles, and therefore the frequency is measured in time. A frequency shift may thus be obtained when the sensor is exposed to different concentrations of GB. Table 1 shows the response results of the sensor. The linear scale is from 0.7 to 3.0 mg/m^3^, and the lowest detecting concentration is 0.1 mg/m^3^. The detection process is reversible and repeatable. Fast responses and notable changes in frequency are obtained within a few seconds, and the detection equilibrium is achieved slowly. The measured responses and recovery times depend on the concentration of GB.

**Table 1:** Responses of the SAW-MIP sensor to different concentrations of GB (Temperature = 25 °C, Relative Humidity = 45%).

Concentration (mg/m^3^)	Frequency Shifts (Hz)	Response Time (s)	Recovery Time (s)
0.1	293	307	45
0.3	430	260	55
0.7	730	220	70
1.0	790	200	81
1.5	925	180	95
2.0	1020	159	103
2.5	1150	148	115
3.0	1300	140	130

In fact, the frequency shift upon gas exposure is due to mass loading of GB molecules adsorbed into the mono[6-deoxy-6-[(mercaptodecamethylene)thio]]-β-CD sensing layer; therefore, the sensing mechanism is based on mass changes in measuring frequency. The measured responses and recovery times suggest that reversible interactions between GB molecules and the sensing layer occur via physisorption or hydrogen binding.

### 3.5. Thermal Stability of the SAW-MIP Sensor

Temperature exerts a direct effect on the operation of all SAW devices. Changes in temperature produce changes in the density of the substrate, including the film and the detected gas, which, in turn, change the velocity of the SAW. No substrate materials that have negligible temperature coefficients are available in practice and the physical properties of the chemically sensitive coating material are often influenced by temperature. In our experiments, the SAW-MIP sensor was evaluated by placing it in a room with temperatures controlled from 10 °C to 50 °C. GB was then detected under different temperatures; the results are listed in Table 2.

**Table 2:** Detection results of the SAW-MIP sensor to GB under different temperatures.

Temperature (ºC)	Frequency Shift (Hz)	Response Time (s)	Recovery Time (s)
10	2503	210	90
20	2127	180	85
30	1438	120	60
40	1024	90	30
50	837	60	10

Our results demonstrate some loss of frequency shift with increasing temperature, and the general trends of response signal, response time and recovery time are fairly obvious. These phenomena may be attributed to molecules with higher temperature being more active than those with lower temperature and therefore being easier to adsorb or desorb by the film. Mono[6-deoxy-6-[(mercaptodecamethylene)thio]]-β-CD may undergo self-exchange with increasing temperature, thereby reducing of effective area of the film. The related adsorption and desorption thermodynamics have studied [25]. Thus, changes in the physical properties of the coating material itself can worsen the effects because of temperature variations, such as a viscoelastic effect.

**Figure 6:**
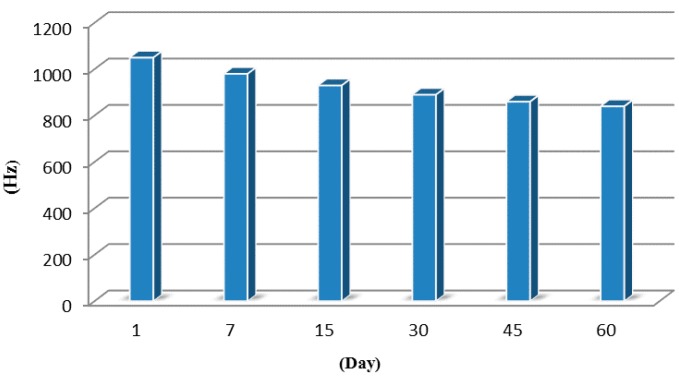
Stability of the sensor after storage for several days.

The sensitivity and reproducibility of the sensor are largely affected by the stability of the organic selective film. To determine the medium stability of the proposed sensor system, the SAW-MIP sensor was kept at room temperature, and GB was detected with the prepared SAW-MIP sensor under the same condition for 60 days. The results indicate that the response frequency of the sensor to GB decreases minimally with increasing storage time. This decrease is fairly obvious within the first 30 days of storage but does not influence the results of the sensor. Over the last 15 days of storage, the sensor maintained steady results, and the advantage of this self-assembly technology was proven (Figure 6).

## 4. Conclusions

In summary, mono[6-deoxy-6-[(mercaptodecamethylene)thio]]-β-CD was successfully prepared by self-assembly MIP technology and tested as a sensing layer in a SAW oscillator sensor for detecting the CWA GB. A one-step approach to assembling a β-cyclodextrin derivative is developed, and the resultant film is stable and relatively uniform. Remarkable linearity, stability, and reversibility, fast responses and high sensitivity were demonstrated.

By preparing a SAW-MIP sensor for GB, a new strategy to extend the proposed method to the detection of nerve agents has been achieved. We conclude that significant imprinting effects could be achieved by preparing imprinted β-cyclodextrin derivatives or other supramolecules. The production of MIPs designed to facilitate GB detection is expected to produce sensors with both selectivity and sensitivity. Based on the results reported here, we believe that MIP presents a rapid and simple alternative method for use in a number of sensing needs.

Our future research work will also involve development of the SAW-MIP with various applications to complement existing technologies, such as biosensing applications. We believe that the advantages presented by these SAW techniques will economically improve gas sensor technology by using these SAW techniques.
